# Characterization of Vegetative Incompatibility in *Morchella importuna* and Location of the Related-Genes by Bulk Segregant Analysis

**DOI:** 10.3389/fmicb.2022.828514

**Published:** 2022-03-07

**Authors:** Hongmei Chai, Ping Liu, Yuanhao Ma, Weimin Chen, Nan Tao, Yongchang Zhao

**Affiliations:** ^1^Biotechnology and Germplasmic Resource Research Institute, Yunnan Academy of Agricultural Sciences, Kunming, China; ^2^Yunnan Provincial Key Laboratory of Agricultural Biotechnology, Kunming, China; ^3^Key Laboratory of Southwestern Crop Resource Germplasm Innovation, Ministry of Agriculture and Rural Affairs, Kunming, China

**Keywords:** allelic polymorphism, ascomycete, barrage, *het* gene, vegetative compatibility group

## Abstract

Vegetative incompatibility (VI) is a widespread phenomenon developed in *Morchella importuna*, a species of ascomycete fungus that is cultivated on a rapidly expanding scale in China. Understanding the genetic bases of this nonself-recognition phenomenon is beneficial for resolving some problems that are associated with the production of this highly prized edible fungus, such as crossbreeding, strain classification, and pathogen transmission. VI is genetically controlled by *het* genes, organized in two different systems, namely allelic and nonallelic. These *het* genes have been well characterized in *Podospora anserina* and *Neurospora crassa.* In this work, putative *het*-homologs were identified in the genome of *M. importuna*, but their low allelic polymorphism in different vegetative compatibility groups (VCGs) suggested that VI in this fungus might not be regulated by these *het* genes. The progeny derived from vegetative compatible parents became a VCG, while the single-ascospore strains from vegetative incompatible parents were divided into four VCGs, and the interaction between the inter-group strains led to the formation of two types of barrages, viz., thin dark line and raised aggregate of hyphae. The Bulk Segregant Analysis confirmed that the genes *mimpvic32* and *mimpvic33* were linked to VI reactions in *M. importuna*; nevertheless, the formation of barrages also occurred between the pairs carrying the same allele of these two genes. In sum, the VI control system in *M. importuna* was complicated, and there were more other allelic or non-allelic VI-related genes.

## Introduction

In filamentous ascomycete fungi, the self/nonself-recognition of conspecific individuals occurs during sexual and asexual phases in the life cycle. At the sexual stage, the recognition is controlled by a single mating-type locus with two idiomorphs termed *MAT1-1* and *MAT1-2* (Turgeon and Yoder, 2000), and individuals of heterothallic species that carry the opposite mating types are sexually compatible and thus could mate to produce offspring (Debuchy and Turgeon, 2006). During the asexual phase, vegetative incompatibility (VI) systems are responsible for recognition and limit the fusion of genetically distinct mycelia. Some forms of demarcation referred to as barrages, including pigmentation, shear belt, and hyphal aggregates, may occur in the zone of contact where two incompatible individuals meet (Cristina and Myron, 2003; Esser, 2016). The molecular genetic basis of VI has been studied in detail in the ascomycete species *Podospora anserina* and *Neurospora crassa* (Paoletti, 2016). The VI is controlled by multiple, unlinked *het* loci (Glass and Kuldau, 1992), which act as allelic and nonallelic systems. In *P. anserina*, incompatibility is caused by the coexpression of *het-s*/*het-S* of the same locus (allelic systems) and controlled by nonallelic systems, including *het-c*/*het-d*, *het-r/het-v*, and *het-c*/*het-e* (Saupe et al., 2001). For *N. crassa*, in addition to nonallelic *pin-c*/*het-c*, and *het*-6/*un*-24, the allelic mating-type idiomorph is involved in incompatibility reaction; however, the expression of a gene named *tol* has been postulated to allow the coexistence of opposite mating-type nuclei during the sexual reproductive phase (Daskalov et al., 2017). The majority of these *het* genes characterize a protein containing a HET domain (Pfam06985), with approximately 150 amino acids (Espagne et al., 2002; Zhao et al., 2015), which plays a fundamental role as the mediator of VI-associated programmed cell death (PCD).

Nonetheless, research indicates that self and nonself-recognition may not be regulated by the *het* loci in some cases or certain species. Micali and Smith (2003) found that in *N. crassa*, barrages were observed to occur between strains that were identical at all major *het* loci, as well as the cases where barrages did not form between strains that had genetic differences at *het-*6, *het-c*, and/or *mat.* In the genome of *Tuber melanosporum*, variable numbers of homologous sequences of VI-like genes were detected, but the expression data of these genes, along with low allelic polymorphism, suggested that they might not mediate VI in *T. melanosporum* (Iotti et al., 2012).

*Morchella importuna* is a great edible fungus belonging to the Ascomycota, Discomycetes, Pezizales, and Morchellaceae. In recent years, the successful field cultivation of *M. importuna* has been achieved in China and gradually expanded (Liu et al., 2017). Therefore, it is important to develop suitable strains by crossbreeding of parents with particular characteristics; meanwhile, the corresponding genetic basis should be studied in-depth. The sexual reproduction of *M. importuna*, a heterothallic organism, requires two haploid strains containing *MAT1-1* and *MAT1-2* idiomorphs (Chai et al., 2017). However, sexually compatible strains often exhibit VI, and there was no correlation between the mating type and vegetative compatibility (Liu et al., 2021). Moreover, VI was ubiquitous among the *M. importuna* strains (Ma et al., 2017; Liu et al., 2021). To date, no *het* genes have been identified in *Morchella* spp.

The objectives of this study were first to describe the barrage types in *M. importuna* and second to determine whether the reported *het* genes regulate VI, and finally to further identify VI-regulated genes through the Bulk Segregant Analysis (BSA) using single-ascospore populations from vegetatively incompatible parents.

## Materials and Methods

### Strains and Isolation of Single Ascospores

Strains YAASMYPL6-3 and YAASMYPL6-1, verified as *MAT1-1* and *MAT1-2* mating-types, respectively (Chai et al., 2017), as well as vegetatively incompatible, were isolated from an ascocarp YAASMYPL6 of *M. importuna*. The crossbreeding of YAASMYPL6-3 and YAASMYPL6-1 was performed by mixing equal parts of the final spawn, followed by the cultivation in a farm in Jinning District (Kunming City, Yunnan Province, China). Among the offspring of the harvest, three ascocarps, namely Y1Y3-1, Y1Y3-2, and Y1Y3-3, with matured ascospores, were selected to develop the single-ascospore populations.

The wild ascocarp Zhao0001 of *M. importuna* was collected from the Yulong County (Yunnan Province, China), and the ascospore print was provided by Dr. Zhao Qi (Kunming Institute of Botany, Academia Sinica). The ascocarps 6611-5 and 6611-7 were obtained from the fields of morel cultivation in the Wuding County (Yunnan Province, China), and their parent strain 6611 was also provided by Dr. Zhao.

For the isolation of single ascospores, a pool of spores with proper initial concentrations was prepared, as reported by Liu et al. (2021). To rule out two or more spores isolated together, each ascospore was collected under a microscope using a small glass capillary tube and then transferred to a 5-cm Petri dish containing potato dextrose agar (PDA). After maintaining 24 h in dark at 22°C, the Petri dish was taken for examination under a dissecting microscope, and the germinated spore colony was transferred to a new Petri dish. Every single-ascospore isolate was numbered with Arabic numerals corresponding to the ascocarp (Table 1) and stored in the Mushroom Center of Yunnan Germplasm Bank of Crops in Yunnan Academy of Agricultural Sciences, Kunming, China.

**TABLE 1:** Single-ascospore populations used in this study.

Single-ascospore populations	Ascocarp	Ascocarp characteristic
Y1Y3-001–Y1Y3-200	Y1Y3-1	Progeny from YAASMYPL6-3 and YAASMYPL6-1 crossed
Y1Y3-201–Y1Y3-400	Y1Y3-2	
Y1Y3-401–Y1Y3-415	Y1Y3-3	
Zhao0001-1–Zhao0001-33	Zhao0001	Wild
6611-5-1–6611-5-13	6611-5	Cultivation
6611-7-1–6611-7-13	6611-7	

### Strain Confrontations

It would be a huge numbers of combinations if one-to-one pairing were performed in the isolates obtained from the ascocarps Y1Y3-1, Y1Y3-2, and Y1Y3-3. Therefore, firstly, confrontational culture was carried out between every single-ascospore strain and the parent strains YAASMYPL6-3 and YAASMYPL6-1, and then the strains were divided into groups based on vegetative compatibility reactions. Secondly, three isolates were randomly chosen from each group to test their confrontational interactions with members of the intra-group and the inter-group, and therefore the vegetative compatibility groups (VCGs) were verified.

According to Chen and Zhang (2010), seven isolates could be paired in all possible combinations in three Petri dishes. For the single-ascospore isolates derived from the ascocarps Zhao0001, 6611-5, and 6611-7, vegetative compatibility assays were performed within the population, and each test was repeated three times in a 9-cm Petri dish.

### Molecular Biology Assays

Total DNA was isolated from the 7-day-old mycelia grown in PDA using the Fungal gDNA Kit (GD2416) (Biomiga, San Diego, CA, United States). Polymerase chain reaction (PCR) was performed in a 25-μL mixture containing 12.5 μL of PCR Mix (Vazyme Biotech Co., Ltd, Nanjing, China), 1 μL of each 10 μM primer, and 1 μL of 10 ng/μL genomic DNA.

Primer pairs for PCR were designed using the software Primer Premier 5.0 (Premier Biosoft International, Palo Alto, CA, United States), and their detailed information, along with amplification conditions, are listed in Supplementary Table 1. The synthesis of primer pairs and sequencing of all PCR products were carried out in Beijing Tsingke Biotechnology. As reported by Chai et al. (2017), the method was used to analyze the mating types of single-ascospore strains.

### Analysis of *het*-Homologs in *Morchella importuna*

Genome databases of *M. importuna*, including YAASMYPL6-3 (GWHBCHL00000000) and YAASMYPL6-1 (GWHBCHM00000000), have been deposited in China National Center for Bioinformation. The VI-related genes in *N. crassa*, *P. anserina*, and *T. melanosporum* were selected according to a study conducted by Mirco et al. (2012) and are listed in Table 2. Searches were run against the genome database of YAASMYPL6-3 using tBLASTn algorithms (Altschul et al., 1997). Genes with an *E*-value of <e^–10^ and >30% identity were selected as targets.

**TABLE 2:** The *het*-homologs in the YAASMYPL6-3 genome of *M. importuna*.

Species	*het*	GenBank number	*Het*-homologues in YAASMYPL6-3	Scaffold in YAASMYPL6-3	Identities (%)	*E*-value
*N. crassa*	*het-c*°*^R^*	AAB48349	GME5750	Scaffold 380	57	0
			GME1887	Scaffold 77	43	3e^–151^
			GME3821	Scaffold 200	44	4e^–146^
	*het-c^PA^*	AAF08294	GME5750	Scaffold 380	57	0
			GME1887	Scaffold 77	42	1e^–149^
			GME3821	Scaffold 200	44	4e^–148^
	*het-c^GR^*	AAF08295	GME5750	Scaffold 380	56	0
			GME1887	Scaffold 77	47	1e^–149^
			GME3821	Scaffold 200	44	5e^–146^
	*pin-c*1	ABC46540	–			
	*Pin-c*2	ABC46541	–			
	*pin-c*3	ABC46542	–			
	*het-6*°*^R^*	Q9UV10	–			
	*un-24*°*^R^*	Q9UW15	GME2544	Scaffold 116	71	0
	*un-24^PA^*	ABF71875	GME2544	Scaffold 116	72	0
	*tol*	AAC64945	–			
*P. anserina*	*het-c2*	AAA20542	GME2473	Scaffold 113	50	1e^–58^
	*het-e*	CAL30215	–			
	*het-d*	CAL30216	–			
	*het-r*	ACM48730	–			
	*het-s*	1718317A	–			
	*het-S*	1718317B	–			
*T. melanosporum*	*tmelhet45*	AFB74445	GME5583	Scaffold 362	35	2e^–34^
			GEM5612	Scaffold 366	30	4e^–31^
	*tmelhet21*	AFB74447	–			

Primer pairs, shown in Supplementary Table 1, were designed to amplify the corresponding fragment in some members of each VCG. Amplified products were prepared for sequencing, and sequence polymorphism analyses were performed using Clustalx 1.83 (Jeannmougin et al., 1998). Sequences of *het*-homologs in some *M. importuna* strains were submitted to GenBank under the accession numbers OL303938–OL303979.

### The Bulk Segregant Analysis

Based on the VI reactions with the parent strains YAASMYPL6-3 and YAASMYPL6-1, the progenies were divided into two VCGs. Sixty strains were selected randomly from each VCG to isolate the genomic DNA, and then DNA samples were pooled by combining equal amounts from each sample to form two DNA bulks. Genomic libraries from two VCG pools and two parents were constructed, and sequencing was performed using the Illumina HiSeq™ 2000 platform by Beijing Biomarker Technologies, and corresponding metadata files were submitted to GenBank under the accession numbers SRR17634835∼SRR17634835. The clean reads of each VCG were aligned to the reference genome YAASMYPL6-3 using Burrows-Wheeler Aligner (BWA v0.7.10) (Li and Durbin, 2009), and multiple read pairs with external coordinates were removed using the Mark Duplicate software of Picard.^1^ Local Realignment, Base Recalibration, Single Nucleotide Polymorphism (SNP), and Insertion-Deletion (InDel) calling were performed using the GATK (McKenna et al., 2010) software. The SnpEff software (Cingolani et al., 2012) was used to annotate SNPs and small InDels based on the GFF files of the reference genome.

The SNPs and small InDels (1–5 bp), supported by fewer than four reads, with multiple genotypes but no polymorphism between the two VCG bulks, were eliminated. After filtering, the ED (Altschul et al., 1997) and SNP-index and InDel-index algorithms (Deng et al., 2006; Ashburner et al., 2010) were performed to obtain significant genetic differences between bulks. The higher the ED value and the closer to 1 the Δ (SNP-index) and Δ (InDel-index) values, the closer the distance to the targeted sites. The ED median + 3 SD of the fitted value at all loci was set as the association threshold. The numbers of association SNPs and small InDels in every scaffold were counted, and the scaffolds enriched with candidate sites were screened by setting the threshold of FDR <0.01. Finally, the candidate scaffolds were obtained by combining ED algorithms and SNP-index algorithms.

## Results

### Homologs of *het* Genes in the Genome of *Morchella importuna*

The tBLASTn searches were run against the YAASMYPL6-3 genome using an *E*-value of <e^–10^ and >30% identity as the filter threshold, and variable numbers of homologous sequences were identified only for *het-c*, *un-24*, *het-c2*, *temelhet45*, and *temelhet21*. No homologues were detected for the *pin-c*, *het-6*, *tol*, *het-e* (*het-d*/*het-r*), and *het-s* (*hetS*) (Table 2).

In the genome YAASMYPL6-3, three predicted genes, namely GME5750, GME1887, and GME3821, were simultaneously identified for each *het-c* gene in *N. crassa*, and all contained a conserved HET-C domain (PF07217). The detected homolog GME2544 showed more than 70% identity to *un-24*°*^R^* and *un-24^PA^*, but the high sequence similarity was only limited to the residues of the first N-terminal ribonucleoside reductase domain (PF02867), with more difference at their C-terminal.

The tBLASTn searches were run using the VI-related genes in *P. anserina* as the query, and only the homolog GME2473 for *het-c2* was identified, which contained the glycolipid transfer protein domain (PF08718). For *het-e*, *het-d*, and *het-r*, more than 50 predicted genes with an *E*-value of <e^–10^ were retrieved, many of which only displayed low similarity at the C-terminal WD-repeat domain, but a few of them showed low identity to the central NACHT (PF05729), and none was characterized by the presence of the N-terminal HET domain. Thus, these retrieved genes were not considered as the candidates.

Two predicted genes GME5583 and GME5612 were detected when the tBLASTn search was performed using the VI-related protein TMELHET45 in *T. melanosporum* as a query. To further investigate the HET-domain-containing genes in the YAASMYPL6-3 genome, tBLASTn searches were also conducted using only HET domain sequences of all selected *het* genes in *N. crassa*, *P. anserina*, and *T. melanosporum*, but no more homologs were identified.

### Mycelial Interactions in *Morchella importuna*

In this study, almost 10,000 confrontation tests were performed with 100 strains of *M. importuna*. According to the phenotypic features of the antagonistic reaction, barrages could be divided into type I and type II.

**FIGURE 1:**
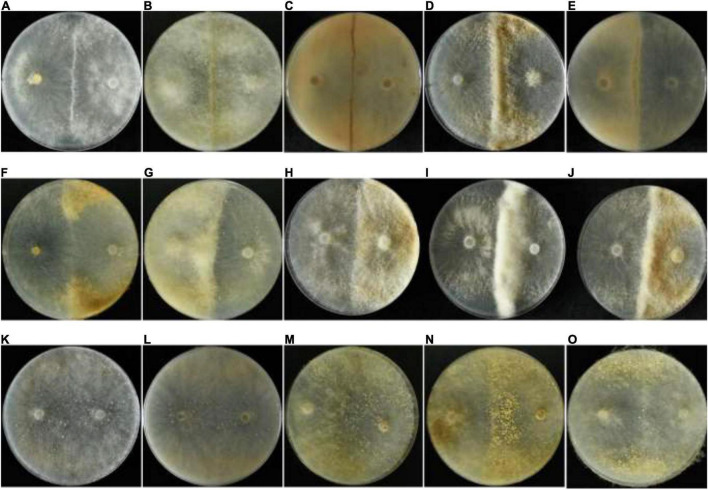
Mycelial interactions. The type I barrage, **(A)** the appearance of an obvious border zone after paired hyphal contact; **(B)** the appearance of an evident dark line after inoculation for 7–10 days; **(C)** a narrow dark line observed from the bottom of the Petri dish. The type II barrage, **(D)** hyphal aggregates formed along the contact zone; **(E)** a wide yellow border observed from the bottom of the Petri dish; **(F)** fan-shaped mats formed between the contact zone and the Petri dish; **(G)** hyphal aggregates formed on one side of the pairs; **(H–J)** three replications for the same pairs. Compatible interactions, **(K)** no demarcation line observed between compatible strains; **(L)** no demarcation observed from the bottom of the Petri dish; **(M)** sclerotia concentrated on one side of the pairs; **(N)** sclerotia concentrated on the contact zone; **(O)** sclerotia concentrated at the angles between the contact zone and the Petri dish.

For type I, a dark reddish-brown line formed a contact zone, and the width was about 0.5–2 mm. After inoculation for 3–4 days, the hyphal branches grew close to each other, and an obvious border-zone infarct could be observed (Figure 1A). At 7–10 days, the pigment was formed in the hyphae, and the border zone turned to an evident dark line, but the width of the zone did not show an obvious change (Figures 1B,C). Except for the dark line formed, the confronting strains grew normally.

In some pairs, the type II barrage occurred, whereas in three cases, mycelia were aggregated to form a dense hyphal mat. First, the dense hyphae could extend out along the interaction zone and develop to barrages ranging from 0.5 to 2 cm (Figure 1D), and a wide, deep yellow-brown border could be observed from the bottom of the Petri dish (Figure 1E). In another case, the dense hyphae formed two prominent fan-shaped mats on one side of the pairs (Figure 1F). Finally, hyphal aggregates appeared on one side of the pairs, and another confronting strain grew normally (Figure 1G). The above-mentioned cases could occur simultaneously with three replications for the same pairs (Figures 1H–J), and thus the three antagonistic phenomena were classified as the type II barrage.

Among the strains with vegetative compatibility, lines of demarcation were not observed, and the hyphae from paired interactions grew close to each other when strains were confronted (Figures 1K,L). But sometimes, sclerotia might be concentrated on one side of the confronting strain (Figure 1M), or along the contact zone (Figure 1N), or at the angle between the contact zone and the Petri dish (Figure 1O). These phenomena randomly occurred and were not specific to certain strains.

### The Determination of Vegetative Compatibility Groups

**FIGURE 2:**
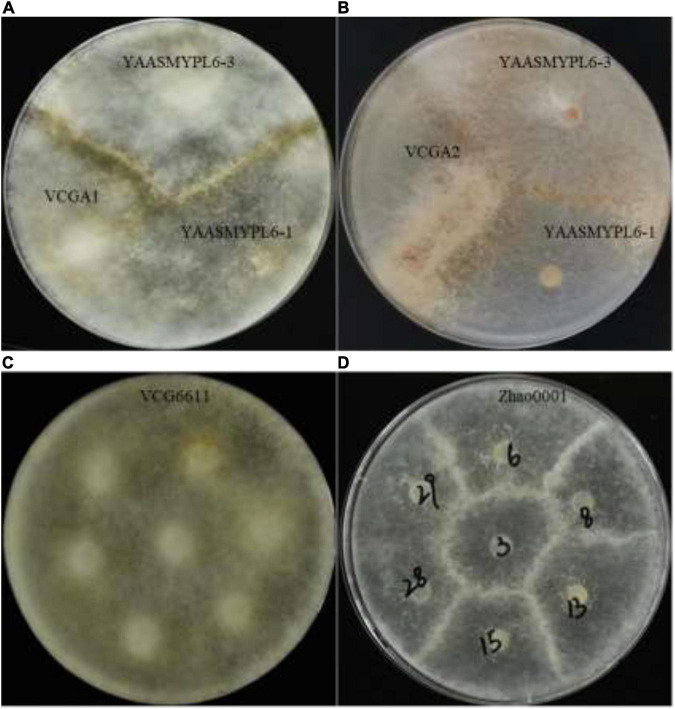
The confrontation test against strains. **(A)** The interaction between the VCGA1 strain and the parent strains. **(B)** The interaction between the VCGA2 strain and the parent strains. **(C)** Interactions between seven strains from the VCG6611 population. **(D)** Interactions between seven strains from the Zhao0001 population; Zhao0001-28 and Zhao0001-29 strains were compatible.

A total of 415 single-ascospore strains were isolated from the ascocarps Y1Y3-1, Y1Y3-2, and Y1Y3-3. By confrontation assays with the parent strains YAASMYPL6-3 and YAASMYPL6-1, the progenies were divided into four VCGs. A total of 118 strains showed the type I barrage with YAASMYPL6-3, while with YAASMYPL6-1, they compatibly grew and were classified as VCGA1 (Figure 2A). Ninety-nine strains exhibited type I barrage with YAASMYPL6-3 and type II barrage with YAASMYPL6-1, and were then categorized into VCGA2 (Figure 2B). Conversely, the type I barrage with YAASMYPL6-1 occurred for 96 VCGB1 strains, which had the vegetative compatibility with YAASMYPL6-3, whereas 83 identified VCGB2 strains showed type I barrage with YAASMYPL6-1 and type II barrage with YAASMYPL6-3. Due to slow growth, confrontation tests were not done with the other 19 strains.

Three strains were selected randomly from each VCG to carry out confrontation assays with intra-group and inter-group strains to assess vegetative compatibility relationships among the four groups. The results suggested that the type I barrage occurred between VCGA (VCGA1 and VCGA2) and VCGB (VCGB1 and VCGB2), while type II barrage was observed between VCGA1 and VCGA2 and between VCGB1 and VCGB2; meanwhile, the vegetative compatibility relationship among the members within a group was further confirmed. The results of confrontation tests between the selected 12 strains are presented in Table 3.

**TABLE 3:** Results of the confrontation experiments between the 12 selected strains.

		VCGA1			VCGA2			VCGB1			VCGB2		
		282	336	346	039	068	088	065	212	134	004	005	220
VCGA1	282	–											
	336	–	–										
	346	–	–	–									
VCGA2	039	II	II	II	–								
	068	II	II	II	–	–							
	088	II	II	II	–	–	–						
VCGB1	065	I	I	I	I	I	I	–					
	212	I	I	I	I	I	I	–	–				
	134	I	I	I	I	I	I	–	–	–			
VCGB2	004	I	I	I	I	I	I	II	II	II	–		
	005	I	I	I	I	I	I	II	II	II	–	–	
	220	I	I	I	I	I	I	II	II	II	–	–	–

*The prefix “Y1Y3-” was omitted from all the strain numbers. –, vegetative compatibility; I, type I barrage; II, type II barrage.*

All 351 [C(27, 2) = (27 × 26) / 2 = 351] combinations, made between the 27 strains 6611-5-1–6611-5-13, 6611-7-1–6611-7-13, and 6611, were analyzed. The results demonstrated that they were all vegetatively compatible and could be classified into a VCG, named VCG6611 (Figure 2C). All 630 [C(36, 2) = (36 × 35) / 2 = 630] combinations of the confrontation tests with 36 strains Zhao0001-1–Zhao0001-33, YAASMYPL6-1, YAASMYPL6-3, and 6611 were made, and part of the results presented in Figure 2D. Except the combination of Zhao0001-28 and Zhao0001-29 was vegetative compatible, the other 629 combinations were vegetative incompatible and exhibited the type I barrage, as showed in Figure 2D between the strains Zhao0001-3 and Zhao0001-6, Zhao0001-8, Zhao0001-13, and Zhao0001-15.

Mating-type identifications of some strains randomly selected from each VCG were performed, and the results showed the presence of *MAT1-1* and *MAT1-2* strains in every VCG.

### Analysis of *het*-Homologs Polymorphisms

Seven candidate sequences of *het*-homologs were derived from the genome YAASMYPL6-3, and the corresponding primer pairs, P24 to P30, were designed (Supplementary Table 1). DNA sequence polymorphism analysis was performed with 25 strains, including the members from VCGA1, VCGA2, VCGB1, VCGB2, VCG6611, as well as the Zhao0001 population (Supplementary Table 2).

The sequence alignment analysis of PCR products indicated that there were low levels of allelic polymorphism of these homologs among the members belonging to different VCGs. Amplified sequences of 25 strains were identical when the primer pairs P24, P25, P27, P28, and P29 were used. With the primer pair P26, four SNP loci were detected in 25 amplified PCR products; however, they were not distributed mainly in a particular VCG. In addition, two SNP loci were located in the introns, while the other two were non-synonymous coding. In the products amplified with the primer pair P30, two SNP loci were detected only in four Zhao0001 strains, and only one SNP locus was synonymous coding.

In conclusion, for each *het*-homolog, there were no allelic polymorphisms between the VCGs in this study, and they might not be able to regulate the VI in *M. importuna*.

### Location of Vegetative Incompatibility-Related Genes by Bulk Segregant Analysis

**FIGURE 3:**
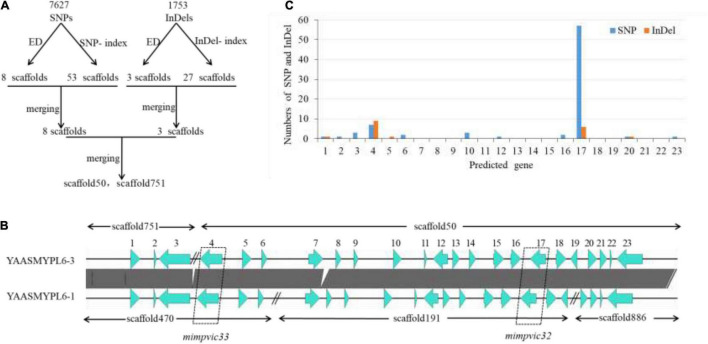
Location of VI-related genes by Bulk Segregant Analysis. **(A)** Overview of the analysis workflow for screening the candidate scaffolds. **(B)** The comparative analysis of the YAASMYPL6–1 and YAASMYPL6–3 genomes showed that the scaffolds 50 and 751 were closely connected. **(C)** Comparison of the SNPs and InDels distributed in 23 predicted genes.

Genomic libraries of VCGA and VCGB bulks and two parents were constructed, and sequencing was performed. A total of 15,752 SNPs and 4,140 InDels were detected by the alignment of each sample to the reference genome YAASMYPL6-3. After filtering, 7,627 SNPs and 1,753 InDels showed high quality for further association analysis. The Euclidean Distance (ED) algorithm was applied to measure allele segregation and to identify the linked scaffolds based on the SNPs between the two bulks. Using the median + 3 SD of the fitted value at all loci as the cut-off, the association SNPs in every scaffold were identified, and eight scaffolds (FDR <0.01) which significantly enriched candidate SNPs were screened out (Supplementary Table 3). Meanwhile, the SNP-index algorithm was performed to analyze significant differences in genotype frequencies between the two bulks, and the numbers of association SNPs in every scaffold were included in Supplementary Table 4 and 53 linked scaffolds (FDR <0.01) were identified. Eight overlapping scaffolds using these two algorithms represented the target scaffolds based on SNP analysis (Table 4). Similarly, small InDel association analysis was carried out using the ED and InDel-index algorithms, then 3 and 27 scaffolds (FDR <0.01) which enriched candidate sites were detected respectively (Supplementary Tables 5, 6). Three scaffolds could be found in the intersection of these two algorithms, which were the target scaffolds based on small InDel analysis (Table 4). Ultimately, these target scaffolds intersected to obtain the candidate scaffolds 50 and 751 (Table 4 and Figure 3A).

**TABLE 4:** The target scaffolds based on SNP and InDel association analysis.

Target scaffolds based on SNP association analysis				Target scaffolds based on InDel association analysis			
Scaffold	AllSNP	AssoSNP_ED	AssoSNP_SNP-index	Scaffold	AllInDel	AssoInDel_ED	AssoInDel_InDel-index
Scaffold50*	179	179	179	Scaffold50	43	42	43
Scaffold751*	44	44	44	Scaffold751	10	10	10
Scaffold40	49	12	49	Scaffold1303	3	3	3
Scaffold613	12	6	10				
Scaffold1388	12	4	12				
Scaffold1798	8	7	7				
Scaffold1876	6	4	6				
Scaffold2066	4	3	4				

*AllSNP, the numbers of all SNPs in the scaffold; AssoSNP_ED, the numbers of association SNPs in the scaffold using the ED algorithm; AssoSNP_SNP-index, the numbers of association SNPs in the scaffold using the SNP-index algorithm; AllInDel, the numbers of all small InDels in the scaffold; AssoInDel_ED, the numbers of association InDels in the scaffold using the ED algorithm; AssoInDel_InDel-index, the numbers of association InDels in the scaffold using the InDel-index algorithm.*

*The final candidate scaffolds.

The analysis of the YAASMYPL6-1 and YAASMYPL6-3 genomes showed that the scaffolds 50 and 751 were closely connected, and they harbored 23 predicted genes (Figure 3B) of which annotation were listed in Supplementary Table 7. Among them, gene 4, 12, 16, and 17 were non-synonymous mutant genes between the two parents. Further analysis revealed that genes 12 and 16 harbored only one and two non-synonymous SNPs respectively, however, gene 4 (*mimpvic32*) and 17 (*mimpvic33*) carried the vast majority of non-synonymous SNPs and InDels (Figure 3C), which were speculated to be VI-related candidate genes.

Sequences of *mimpvic32* in the parental strain YAASMYPL6-1 were defined as A allele *mimpvic32A*, while B allele *mimpvic32B* was in the parental strain YAASMYPL6-3. The lengths of the *mimpvic32A* and *mimpvic32B* sequences were 2,906 and 2,875 bp, respectively, and both contained 8 introns and encoded proteins of 765 amino acids (Table 5). There was a sequence identity of 92.6% between these amino acids, and sequence variances were mainly concentrated on C-terminal regions (Supplementary Figure 1). The BLASTx analysis revealed that the predicted protein contained a CorA-like Mg^2+^ transporter protein domain (PF01544), which is abundant in most bacteria and archaea, and in some eukaryotes (Kehres et al., 1998). The CorA proteins are generally involved in transporting Mg^2+^ across membranes, but the members of the CorA family can also transport other divalent cation such as cobalt and nickel (Niegowski and Eshaghi, 2007). Besides, the CorA family includes the MRS2 protein from yeast that is thought to be an RNA splicing protein (Wiesenberger et al., 1992).

**TABLE 5:** Allelic variation of the VI-related candidate genes *mimpvic32* and *mimpvic33*.

Strain	*mimpvi32*					*mimpvi33*				
	Allele type	Intron number	DNA (bp)	Amino acids (aa)	GenBank accession number	Allele type	Intron number	DNA (bp)	Amino acids (aa)	GenBank accession number
YAASMYPL6-1	A	8	2,906	773	OL303981	A	2	4,078	1,311	OL303983
YAASMYPL6-3	B	8	2,875	773	OL303980	B	2	4,117	1,324	OL303982
Zhao0001-28	B	8	2,875	773	OL303980	C	2	4,090	1,315	OL303984

Sequences of *mimpvic33* in the parental strains YAASMYPL6-1 was defined as A allele *mimpvic33A*, whereas B allele *mimpvic33B* was found in the strain YAASMYPL6-3. The lengths of the sequences *mimpvic33A* and *mimpvic33B* were 4,078 and 4,117 bp, and both contained 2 introns, with encoded proteins of 1311 and 1324 amino acids (Table 4), respectively. Compared with *mimpvic33B*, *mimpvic33A* had 4 amino acids difference, and 4-aa and 9-aa were missed. There was a 98.7% amino acid sequence similarity between the two alleles (Supplementary Figure 2). The BLASTx analysis revealed that the predicted protein showed 26.5% amino acid similarity to the hypothetical protein of white truffle *T*ube*r borchii* (PUU75087), and had no any conserved protein domain family been found.

### Allelic Polymorphisms of *mimpvic32* and *mimpvic33*

Primer pairs P32 and P33 were designed to amplify the genes *mimpvic32* and *mimpvic33* (Supplementary Table 1). To verify allelic polymorphisms in different VCG populations, except for 15 strains from Zhao0001 population, each 10 single-ascospore strains were randomly selected from VCGA1, VCGA2, VCGB1, VCGB2, and VCG6611 to amplify *mimpvi32* and *mimpvi33*, and the DNA sequence data were analyzed.

Thirty strains from VCGA1, VCGA2, and VCG6611, as well as YAASMYPL6-1, harbored the genes *minpvic32A* and *minpvic33A*. YAASMYPL6-3 and 20 strains from VCGB1 and VCGB2 carried *minpvic32B* and *minpvic33B*. In 15 strains of wild population Zhao0001, no A but C allele of the minpvic33 gene was detected (Supplementary Figure 2), and they had either minpvi32A and minpvi33B, or minpvi32B and minpvi33C (Supplementary Table 8).

For VCGA and VCGB, the phenotypes with vegetative compatibility were consistent with allelic polymorphisms of minpvic32 and minpvic33, but not for VCG6611 and Zhao0001 populations. Ten strains from VCG6611, similar to the strains from VCGA, had *minpvic32A* and *minpvic33A*; however, there was a type I barrage between VCG6611 and VCGA. In the wild population Zhao0001, though Zhao0001-28 and Zhao0001-29 harboring the same minpvic32B and minpvic33C were compatible, the other strains, such as Zhao0001-6, Zhao0001-8, Zhao0001-15, Zhao0001-21, and so on, carrying the same alleles displayed VI. Therefore, we speculate that besides *minpvic32* and *minpvic33*, there were other genes involved in the regulation of VI in *M. importuna*.

## Discussion

Vegetative incompatibility, a form of nonself allorecognition, is ubiquitous in filamentous fungi and may limit the spread of viruses (Zhang et al., 2014) or deleterious genetic elements and prevent the plundering of resources (Pál1 et al., 2007). VI has been widespread in *Morchella Mes*-19, *M*orchella *sextelata*, and *M. importuna* (Liu et al., 2021). According to Yu and Zhao (2020), pairing the strains of *M. importuna* with 100% genetic similarity based on the ISSR (inter-simple sequence repeats) analysis resulted in the occurrence of antagonistic actions. Ma et al. (2017) isolated 15 strains from the cultivation regions of *M. importuna* in Nanyang city of China, and the results of confrontation tests showed that 101 out of 105 combinations were antagonistic. Our data also indicated that VI was a universal phenomenon among the strains of *M. importuna*, and the strains from different populations presented VI.

All 26 single-ascospore strains from the cultured ascocarps 6611-5 and 6611-7 were compatible and they formed a VCG with the parent strain 6611. We deduced that the two nuclei with the opposite mating types in strain 6611 were vegetatively compatible, thus indicating that all progenies from strain 6611 would be assigned to a VCG. However, the offspring from vegetatively incompatible parents was more complicated. The single-ascospore strains from incompatible parents were divided into four VCGs, and different manifestation of incompatibility exhibited between the inter-group strains. The VI interactions of VCGA1 and VCGB1 were consistent with those of the parental strains YAASMYPL6-1 and YAASMYPL6-3, respectively, whereas the VCGA2 and VCGB2 presented new phenotypic characteristics and showed type I or type II barrages with the parents. In *M*orchella *esculenta*, no demarcation formed when monoascospore cultures from single ascus were paired, but horizontal or longitudinal fluffy zone occurred when confrontation tests were preformed between eight ascospore isolates of two asci of same ascocarp (Singh et al., 2006). The horizontal and longitudinal fluffy zone were similar to the type II barrages in this study. We speculated that the strains in VCGA1, VCGA2, VCGB1, and VCGB2 originated from different asci respectively, and among which distinct genetic recombination events occurred.

Our analyses indicated that in the genome of *M. importuna*, homologous sequences were detected only for *het-c*, *un-24*, *het-c2*, and two HET domain-containing genes, but they did not exhibit allelic polymorphisms between different VCGs. Moreover, all of them were not located in the candidate scaffolds obtained by the BSA. We speculate that these *het* genes may not control the VI reaction in *M. importuna.* The results of BSA allowed us to screen out the two VI-related *mimpvic32* and *mimpvic33* loci, with two and three alleles, respectively. However, with BSA results, we could only look for VI-related alleles, while the genes mediating nonallelic interactions could not be detected.

The members from VCGA1, VCGA2, and VCG6611, as well as YAASMYPL6-1, all carried *mimpvic32A* and *mimpvic33A*, but between the pairs, different barrage types were described. The strains of the zhao0001 population carried the same alleles of the *mimpvic32* and *mimpvic33* loci and also showed VI. These phenomena indicated that the VI control system in *M. importuna* was complicated, and there were more other allelic or nonallelic VI-related genes.

In *N. crassa*, mating-type locus acts as a vegetative-incompatibility locus, and the vegetative coexpression of opposite mating types is lethal (Beadle and Coonradt, 1944; Garnjobst, 1953). However, mating type-associated VI was not found in most filamentous ascomycetes (Saupe, 2000). In this study, the strains from the same VCG could be *MAT1-1* or *MAT 1-2*, and pairing the strains of the opposite mating type, with both vegetatively compatible and incompatible reactions, would occur. Therefore, in *M. importuna*, mating-type locus does not participate in the regulation of VI. But during the sexual phase, the mechanisms that regulate the ability of two vegetatively incompatible nuclei to coexist in the cytoplasm and undergo karyogamy are not yet clear.

## Conclusion

The study indicated that VI was widespread in *M. importuna* and two types of barrages formed in the antagonistic reaction. Self/nonself-recognition in this fungus might not be regulated by the reported het genes, whereas, two genes *mimpvic32* and *mimpvic33* were associated with the VI reactions.

## Data Availability Statement

The datasets presented in this study can be found in online repositories. Raw sequences are deposited in the Sequence Read Archive under Bioproject PRJNA797544. The names of the repository/repositories and accession number(s) can be found in the article/Supplementary Material.

## Author Contributions

PL, YM, WC, and NT designed and performed the experiments, and analyzed the data. YZ conceived and designed the experiments. HC conceived and designed the experiments, and wrote the original manuscript. All authors contributed to the article and approved the submitted version.

## Conflict of Interest

The authors declare that the research was conducted in the absence of any commercial or financial relationships that could be construed as a potential conflict of interest.

## Publisher’s Note

All claims expressed in this article are solely those of the authors and do not necessarily represent those of their affiliated organizations, or those of the publisher, the editors and the reviewers. Any product that may be evaluated in this article, or claim that may be made by its manufacturer, is not guaranteed or endorsed by the publisher.

## Footnotes

1
http://sourceforge.net/projects/picard/
