# Supplementary material for: Characterization of Vegetative Incompatibility in Morchella importuna and Location of the Related-Genes by Bulk Segregant Analysis
Source: Front Microbiol. 2022 Mar 7;13:828514. doi: 10.3389/fmicb.2022.828514 (PMC8940278; doi:10.3389/fmicb.2022.828514)
Supplement: Supplementary file 2 [file Image_1.pdf]

```
                 10        20        30        40        50        60        70        80        90       100
                 ....|....|....|....|....|....|....|....|....|....|....|....|....|....|....|....|....|....|....|....|
mimpvic32A       MATGNQTSSQQSPPRQAGGNQSPRIRG-PIPTIPTVTITYVAPTVPTVQKVSTGIPYQWVTPGTVINTAFLWNNPAVVGDLEFWVESRRAKLSDDEKARL
mimpvic32B       MATGNQTSSQQSPPRQAGGNQSPRINGGPIPTIPTVTITYVAPTVPTVQKVSTGIPYQWVTPGTVINTAFLWNNPAVVGDLEFWVESRRAKLSDDEKARL
Clustal Consensus *************************.* ************************************************************************

                110       120       130       140       150       160       170       180       190       200
                 ....|....|....|....|....|....|....|....|....|....|....|....|....|....|....|....|....|....|....|
mimpvic32A       DSIDETMKALEDPAWYYWPTWDQALQCGRHGTEQELRRSIPTLMWTDEQPMSEIYTPIAQILQDAIKKNFPLEGNYWVAFRDRPLDNLGFIENLSLHKII
mimpvic32B       DSIDETMKALEDPAWYYWPTWDQALQCGRHGTEQELHRSIPTLMWTDEQPMSEIYTPIAQILQDAIKKNFPLEGNYWVAFRDRPLDNLGFIENLSLHKII
Clustal Consensus ************************************:***************************************************************

                210       220       230       240       250       260       270       280       290       300
                 ....|....|....|....|....|....|....|....|....|....|....|....|....|....|....|....|....|....|....|
mimpvic32A       YFVGEKGGGTDFDYKRWPKRGNSYRHIMFPLNKYLWIRKLDDLFKDIHLPPPYGPVLTSDPFRASMCRGYFNTFAKPVVECQPSVGKAYKITISMPILHV
mimpvic32B       YFVGEKGGGTDFDYKRWPKRGNSYRHIMFPLTKYLWIRKLDDLFKDIHLPPPHGPVLNSDPFRASMCRGYFNTFAKPVVECQPPVGKAYKITISMPILHV
Clustal Consensus *******************************.********************:****.*************************.****************

                310       320       330       340       350       360       370       380       390       400
                 ....|....|....|....|....|....|....|....|....|....|....|....|....|....|....|....|....|....|....|
mimpvic32A       TLGSKLIPKKTAERVLRHHPSFLRAPGYEYAETYIDQDGKEQPKPGAWIYGSALDHTGVGFSAASDYDGSTSGTPPTVFTGRTLFEYHHPKMDALILDLR
mimpvic32B       TLGSKLIPKKTAERVLRHHPSFLRAPGYEYAETYIDQDGKEQPKPGAWIYGSALDHTGVSFSAASDYDGSTSGTPPTVFTGRTLFEYHHPKMDALTLDLR
Clustal Consensus ***********************************************************.*********************************** ****

                410       420       430       440       450       460       470       480       490       500
                 ....|....|....|....|....|....|....|....|....|....|....|....|....|....|....|....|....|....|....|
mimpvic32A       QTMRRGNRGHYDDDEFLVVEQLWAVIIPTKETLITFQSMKPAVIPGQVGGFNSYMFEPNKGMDVTPQGPVHGFFIYFADLVLLKHYHILEVFKEGTAGLN
mimpvic32B       QTMRRGNRGHYDDDEFLVVEQLWAVIIPTKETLITFQSMKKAVIPGQVGGFNSYMFEPSKGADVTPQGPVHGFLIYFADLVLLQHYHILEAFKEGTAALN
Clustal Consensus **************************************** *****************.** ***********:*********:******.******.**

                510       520       530       540       550       560       570       580       590       600
                 ....|....|....|....|....|....|....|....|....|....|....|....|....|....|....|....|....|....|....|
mimpvic32A       FRVAHKPITDDELVADCNSVISEVAIILEITKKQMEILDIIKEATVDNTLRPEELAATNEFYKYMMARFEIMVAAFEDIGAEAKQAQSLLFQLLNTEAAK
mimpvic32B       FRVAHKPIADEELVADCNAVISEVAIILEITKKQMEILGMIKEAAIDNTLRPEEFSAANEFYKYMMGRFEIVVATFEDIGAEAKQAQDLLFQLLNIEASK
Clustal Consensus ********:*:*******:*******************.:****::********::*:********.****:**:************.******* **:*

                610       620       630       640       650       660       670       680       690       700
                 ....|....|....|....|....|....|....|....|....|....|....|....|....|....|....|....|....|....|....|
mimpvic32A       QQAIMAEQQAINARQAAEETAVQLELSKEAAKHTAVQLELAKQSTEQNKSFLVFTIITTIFLPLSFFTSYFGMNTVDIRDMEWDQRYFWKTAGPSSAMII
mimpvic32B       KQVAMAIQQEIIARQTAEETAVQLELAKQAAEQAAFQVKLAKQSTEQNKSFLVFTIITTIFLPLSFFTSYFGMNTVDIRDMEWDQRYFWKTAGPSSAMII
Clustal Consensus :*. ** ** * ***:**********:*:**:::*.*::*************************************************************

                710       720       730       740       750       760
                 ....|....|....|....|....|....|....|....|....|....|....|....|....
mimpvic32A       LVVMCWAFRAQMKRGIGILFRSKLKPAQSDEESAIGSLEKSGQATGKRGKSSWNPFGRGVDKND
```

```
mimpvic32B         LVVMCWAFRAQMKRGIDIALQSKVKSAQIDEESAIGSPEKIGQVTGKRGKSSWNSFGRDVDKND
Clustal Consensus  ****************.* ::**:*.** ******** ** **.**********.***.*****
```

Supplementary Figure 1. Amino acid alignment of *mimpvic32* alleles in strain YAASMYPL6-1 (*mimpvic32A)* and in strain YAASMYPL6-3 (*mimpvic32B)* performed using Clustalx 1.83.
