# Supplementary material for: Characterization of Vegetative Incompatibility in Morchella importuna and Location of the Related-Genes by Bulk Segregant Analysis
Source: Front Microbiol. 2022 Mar 7;13:828514. doi: 10.3389/fmicb.2022.828514 (PMC8940278; doi:10.3389/fmicb.2022.828514)
Supplement: Supplementary file 3 [file Image_2.pdf]

```
                           10        20        30        40        50        60        70        80        90       100
                   ....|....|....|....|....|....|....|....|....|....|....|....|....|....|....|....|....|....|....|....|
mimpvic33B         MTDLTWIICTLLALNFVRASHFGRFSPEQTGQNLGRITVYAFISICLHALLPWIQLDTQGSTYWYICRLSFATLIILNGGLYYIHRLPFTGRFILGLSWT
mimpvic33C         MTDLTWIICTLLALNFVRASHFGRFSPEQTGQNLGRITVYAFISICLHALLPWIQLDTQGSTYWYICRLSFATLIILNGGLYYIHRLPFTGRFILGLSWT
mimpvicA           MTDLTWIICTLLALNFVRASHFGRFSPEQTGQNLGRIIVYAFISICLHALLPWIQLDTQGSTYWYICRLSFATLIILNGGLYYIHRLPFTGRFILGLSWT
Clustal Consensus  ************************************* **************************************************************

                          110       120       130       140       150       160       170       180       190       200
                   ....|....|....|....|....|....|....|....|....|....|....|....|....|....|....|....|....|....|....|....|
mimpvic33B         PNQGIGTPGFVDPQNKVDTEANIALRAARRNIPIALELQEKELFDTVRLEEECRRVNPLYSPQDVQRSGYHAYDFLEYVKYRRPRPRPGAEPRIALTDPS
mimpvic33C         PNQGIGTPGFVDPQNKVDTEANIALRAARRNIPIALELQEKELFDTVRLEEECRRVNPLYSPQDVQRSGYHAYDFLEYVKYRRPRPRLGAEPRIALTDPS
mimpvicA           PNQGIGTPGFVDPQNKVDTEANIALRAARRNIPIALELQEKELFDTVRLEEECRRVNPLYSPQDVQRSGYHAYDFLEYVKYRRPRPRLGAEPRIALTDPS
Clustal Consensus  *************************************************************************************** ************

                          210       220       230       240       250       260       270       280       290       300
                   ....|....|....|....|....|....|....|....|....|....|....|....|....|....|....|....|....|....|....|....|
mimpvic33B         EIREVLWWAEGNRLQCHNSFATAPGSNGSLPAFENESVIFTNCVSVERPEQVAPAINLIKQTLRNLPYYTTREPSRNQFKYAVGIDVEFLEFPTEFYRQM
mimpvic33C         EIREVLWWAEGNRLQCHNSFATAPGSNGSLPAFENESVIFTNCVSVERPEQVAPAINLIKQTLRNLPYYTTREPSRNQFKYAVGIDVEFLEFPTEFYRQM
mimpvicA           EIREVLWWAEGNRLQCHNSFATAPGSNGSLPAFENESVIFTNCVSVERPEQVAPAINLIKQTLRNLPYYTTREPSRNQFKYAVGIDVEFLEFPTEFYRQM
Clustal Consensus  ****************************************************************************************************

                          310       320       330       340       350       360       370       380       390       400
                   ....|....|....|....|....|....|....|....|....|....|....|....|....|....|....|....|....|....|....|....|
mimpvic33B         VMSVRGLGDRSFRQPNGYDIGPDGNIILDGTARVETEGYYPLVEDQYLASCMSIAVDKVLVVNFHILHMIRNIETADQLQQFAAIFEQIIFDTTIIKVWF
mimpvic33C         VMSVRGLGDRSFRQPNGYDIGPDGNIILDGTARVETEGYYPLVEDQYLASCMSIAVDKVLVVNFHILHMIRNIETADQLQQFAAIFEQIIFDTTIIKVWF
mimpvicA           VMSVRGLGDRSFRQPNGYDIGPDGNIILDGTARVETEGYYPLVEDQYLASCMSIAVDKVLVVNFHILHMIRNIETADQLQQFAAIFEQIIFDTTIIKVWF
Clustal Consensus  ****************************************************************************************************

                          410       420       430       440       450       460       470       480       490       500
                   ....|....|....|....|....|....|....|....|....|....|....|....|....|....|....|....|....|....|....|....|
mimpvic33B         NPQQDIIVLDATLDEIYRGTLRRQFNDITGPVPRTPLFRPTWKLRLRGDGQRALFGAPRPAQFNFGPDPIWGTRCPYGFHNNAVNQYGHPCPCRSNNLDL
mimpvic33C         NPQQDIIVLDATLDEIYRGTLRRQFNDITGPVPRTPLFRPTWKLRLRGDGQRALFGAPRPAQFNFGPDPIWGTRCPYGFHNNAVNQYGHPCPCRSNNLDL
mimpvicA           NPQQDIIVLDATLDEIYRGTLRRQFNDITGPVPRTPLFRPTWKLRLRGDGQRALFGAPRPAQFNFGPDPIWGTRCPYGFHNNAVNQYGHPCPCRSNNLDL
Clustal Consensus  ****************************************************************************************************

                          510       520       530       540       550       560       570       580       590       600
                   ....|....|....|....|....|....|....|....|....|....|....|....|....|....|....|....|....|....|....|....|
mimpvic33B         AVIVEFIMRDVGLEPADDDGIQFDRRAWTETRERCSYNDFEQSLLHGDRSSGLLSMLKSSIPVQLALPAPRYCANDCVEECLQWEEPGPACCRACTNYQL
mimpvic33C         AVIVEFIMRDVGLEPADDDGIQFDRRAWTETRERCSYNDFEQSLLHGDRSSGLLSMLKSSIPVQLALPAPRYCANDCVEECLQWEEPGPACCRACTNYQL
mimpvicA           AVIVEFIMRDVGLEPADDDGIQFDRRAWTETRERCSYNDFEQSLLHGDRSSGLLSMLKSSIPVQLALPAPRYCANDCVEECLQWEEPGPACCRACTNYQL
Clustal Consensus  ****************************************************************************************************

                          610       620       630       640       650       660       670       680       690       700
                   ....|....|....|....|....|....|....|....|....|....|....|....|....|....|....|....|....|....|....|....|
mimpvic33B         WQAVSRETKARMFRTFANPSFQTDPVLLGYVAGDVLGISMVLRFLMSTENPRLLLERLRLWSNKPMMGAHNVNFLREPSSPEIPLYIDCKWHATSRLINS
```

```
mimpvic33C          WQAVSRETKARMFRTFANPSFQTDPVLLGYVAGDVLGISMVLRFLMSTENPRLLLERLRLWSNKPMMGAHNVNFLREPSSPEIPLYIDCKWHATSRLINS
mimpvicA            WQAVSRETKARMFRTFANPSFQTDPVLLGYVAGDVLGISMVLRFLMSTENPRLLLERLRLWSNKPMMGAHNVNFLREPSSPEIPLYIDCKWHATSRLINS
Clustal Consensus   ****************************************************************************************************

                             710       720       730       740       750       760       770       780       790       800
                    ....|....|....|....|....|....|....|....|....|....|....|....|....|....|....|....|....|....|....|....|
mimpvic33B          VRLYVDNPSFRRCDMVSWIDDKVLRNSVYRNRRKVEFMQPGRMLYYFLEEFWRRHPKTFGEYVVTKIVATPFRRRRRPAEDDQDDDIGNDGDDGDGDGDG
mimpvic33C          VRLYVDNPSFRRCDMVSWIDDKVLRNSVYRNRRKVEFMQPGRMLYYFLEEFWRRHPKTFGEYVVTKIVATPFRRRRRPAEDDQDDDIGNDGDDGDGDGDG
mimpvicA            VRLYVDNPSFRRCDMVSWIDDKVLRNSVYRNRRKVEFMQPGRMLYYFLEEFWRRHPKTFGEYVVTKIVATPFRRRRRPAEDDQDDDIGNDGDDGDGDGDG
Clustal Consensus   ****************************************************************************************************

                             810       820       830       840       850       860       870       880       890       900
                    ....|....|....|....|....|....|....|....|....|....|....|....|....|....|....|....|....|....|....|....|
mimpvic33B          DGDGGGDGGGDGGGDGGGDGGGGERAEEEVAEPDRWQDEIDAGYVQSQRQFTLDEILRFFDSPSWATMSSDILQMVIGYFELKLMHEYQNDIRRVQTMRE
mimpvic33C          DGDGGGDGGGDGGGDGGGDGGGGERAEEEVAEPDRWQDEIDAGYVQSQRQFTLDEILRFFDSPSWATMSSDILQMVIGYFELKLMHEYQNDIRRVQTMRE
mimpvicA            DGDGDGDGGGDGGGDG----GGGERAEEEVAEPDRWQDEIDAGYVQSQRQFTLDEILRFFDSPSWATMSSDILQMVIGYFELKLMHEYQNDIRRVQTMRE
Clustal Consensus   ****.***********    ********************************************************************************

                             910       920       930       940       950       960       970       980       990      1000
                    ....|....|....|....|....|....|....|....|....|....|....|....|....|....|....|....|....|....|....|....|
mimpvic33B          QLDECLLGLVGLTPADLGIIAAARDVLISTVVAGGHNWFTITRDLLTDTAQDHAVTGGGLFLKPAEENEYRDNWANRPAEIKALLQSLVTLQALNASRER
mimpvic33C          QLDECLLGLVGLTPADLGIIAAARDVLISTVVAGGHNWFTITRDLLTDTAQDHAVTGGGLFLKPAEENEYRDNWANRPAEIKALLQSLVTLQALNASRER
mimpvicA            QLDECLLGLVGLTPADLGIIAAARDVLISTVVAGGHNWFTITRDLLTDTAQDHAVTGGGLFLKPAEENEYRDNWANRPAEIKALLQSLVTLQALNASRER
Clustal Consensus   ****************************************************************************************************

                            1010      1020      1030      1040      1050      1060      1070      1080      1090      1100
                    ....|....|....|....|....|....|....|....|....|....|....|....|....|....|....|....|....|....|....|....|
mimpvic33B          DFVQRLRPMAAQDSRDTLVAATLLGIVPFTMPALRTLLSRTTRATASSELATTDPIILHWALMVLRYNRPHTRDRSRTLAERFFDITYSPTNFYMPLLRR
mimpvic33C          DFVQRLRPMAAQDSRDTLVAATLLGIVPFTMPALRTLLSRTTRATASSELATTDPIILHWALMVLRYNRPHTRDRSRTLAERFFDITYSPTNFYMPLLRR
mimpvicA            DFVQRLRPMAAQDSRDTLVAATLLGIVPFTMPALRTLLSRTTRATASSELATTDPIILHWALMVLRYNRPHTRDRSRTLAERFFDITYSPTNFYMPLLRR
Clustal Consensus   ****************************************************************************************************

                            1110      1120      1130      1140      1150      1160      1170      1180      1190      1200
                    ....|....|....|....|....|....|....|....|....|....|....|....|....|....|....|....|....|....|....|....|
mimpvic33B          VIKRATPITRYERSQPGRHYLVLRADNRALQRLRDERAGRIQPQRPPRGDVVTMRGAGVPQVIEVLQEERGWGVDGGDAEFDGDDDDGDADDWCNGTGVE
mimpvic33C          VIKRATPITRYERSQPGRHYLVLRADNRALQRLRDERAGRIQPQRPPRGDVVTMRGAGVPQVIEVLQEERGWGVDGGDAEFDGDGDDGDADDWCNGTGVE
mimpvicA            VIKRATPITRYERSQPGRHYLVLRADNRALQRLRDERAGRIQPQRPPRGDVVTMRGAGVPQVIEVLQEERGWGVDGGDAEFDGDGDDGDADDWCNGTGVE
Clustal Consensus   ************************************************************************************.***************

                            1210      1220      1230      1240      1250      1260      1270      1280      1290      1300
                    ....|....|....|....|....|....|....|....|....|....|....|....|....|....|....|....|....|....|....|....|
mimpvic33B          QLWDATDDLPIWDANADEDEEVPEPEEEPAEELPAEDAAGEPPAEDADGFRGDWNTDLPPPAVRISSLFFEINDEAAPVVQQAQGLSDSESDLESDFDGE
mimpvic33C          QLWDATDDLPIWDANADEDEEVPEPEEEPAEELP---------AEDADGFRGDWNTDLPPPAVRISSLFFEINDEAAPVVQQAQGLSDSESDLESDFDGE
mimpvicA            QLWDATDDLPIWDANADEDEEVPEPEEEPAEELP---------AEDADGFRGDWNTDLPPPAVRISSLFFEINDEAAPVVQQAQGLSDSESDLESDFDGE
Clustal Consensus   **********************************         *********************************************************
```

```
                    1310       1320
             ....|....|....|....|...
mimpvic33B        EDMSVEKAQFLRMLQETLNIWCN
mimpvic33C        EDMSVEKAQFLRMLQETLNIWCN
mimpvicA          EDMSVEKAQFLRMLQETLNIWCN
Clustal Consensus ***********************
```

Supplementary Figure 2. Amino acid alignment of *mimpvic33* alleles in strain YAASMYPL6-1 (*mimpvic33A*), in strain YAASMYPL-3 (*mimpvic33A*) and in strain Zhao0001-28 (*mimpvic33A*) performed using Clustalx 1.83.
